# *N*1-methylnicotinamide impairs gestational glucose tolerance in mice

**DOI:** 10.1530/JME-23-0126

**Published:** 2024-01-08

**Authors:** Xiaojing Wei, Yutian Tan, Jiaqi Huang, Ximing Dong, Weijie Feng, Tanglin Liu, Zhao Yang, Guiying Yang, Xiao Luo

**Affiliations:** 1 Department of Physiology and Pathophysiology, School of Basic Medical Sciences, Xi’an Jiaotong University Health Science Center, Xi’an, China; 2 Key Laboratory of Environment and Genes Related to Diseases, Ministry of Education of China, Xi’an Jiaotong University, Xi’an, China; 3 Institute of Neuroscience, Translational Medicine Institute, Xi’an Jiaotong University Health Science Center, Xi’an, China; 4 Institute of Basic Medicine, School of Medicine, Tsinghua University, Beijing, China; 5 Department of Obstetrics and Gynecology, The First Affiliated Hospital of Xi'an Jiaotong University, Xi'an, China

**Keywords:** gestational diabetes mellitus, glucose tolerance, mice, NAD+ metabolism, *N*1-methylnicotinamide

## Abstract

*N*1-methylnicotinamide (MNAM), a product of methylation of nicotinamide through nicotinamide *N*-methyltransferase, displays antidiabetic effects in male rodents. This study aimed to evaluate the ameliorative potential of MNAM on glucose metabolism in a gestational diabetes mellitus (GDM) model. C57BL/6N mice were fed with a high-fat diet (HFD) for 6 weeks before pregnancy and throughout gestation to establish the GDM model. Pregnant mice were treated with 0.3% or 1% MNAM during gestation. MNAM supplementation in CHOW diet and HFD both impaired glucose tolerance at gestational day 14.5 without changes in insulin tolerance. However, MNAM supplementation reduced hepatic lipid accumulation as well as mass and inflammation in visceral adipose tissue. MNAM treatment decreased GLUT4 mRNA and protein expression in skeletal muscle, where NAD+ salvage synthesis and antioxidant defenses were dampened. The NAD+/sirtuin system was enhanced in liver, which subsequently boosted hepatic gluconeogenesis. GLUT1 protein was diminished in placenta by MNAM. In addition, weight of placenta, fetus weight, and litter size were not affected by MNAM treatment. The decreased GLUT4 in skeletal muscle, boosted hepatic gluconeogenesis and dampened GLUT1 in placenta jointly contribute to the impairment of glucose tolerance tests by MNAM. Our data provide evidence for the careful usage of MNAM in treatment of GDM.

## Introduction

Gestational diabetes mellitus (GDM) is the most common pregnancy complication in the context of the global obesity epidemic (Wang *et al.* 2022). GDM begets various adverse perinatal outcomes and increases the long-term risks of type 2 diabetes and childhood obesity in both mother and offspring (McIntyre *et al.* 2019). The prevalence of GDM is rapidly rising and so is the great need for prevention and treatment (Lee *et al.* 2018). Substantial studies have reported that lifestyle modifications and insulin administration are preferred and effective strategies for treating hyperglycemia in pregnancy (Buchanan *et al.* 2012, Brown *et al.* 2017). Administration of metformin, probiotics, and vitamin D are the most common pharmacological interventions that have been assessed (Rasmussen *et al.* 2020). However, no oral agents appear to be universally effective for the prevention of GDM or safe for mother, developing fetus and offspring. Therefore, seeking safe, effective, and easy-to-administer new treatments for reducing GDM incidence is of great importance.

*N*1-methylnicotinamide (MNAM) is the primary metabolite of vitamin B3 (nicotinamide) by the enzyme nicotinamide *N*-methyltransferase (NNMT) (Pissios 2017). Plasma MNAM level is positively correlated with body mass index (BMI), and inversely correlated with insulin sensitivity in men and women (Kannt *et al.* 2015, Liu *et al.* 2015). Metabolomic analyses reveal elevated levels of urinary MNAM in *db/db* male mice and obese Zucker rats, suggesting increased NNMT activity in obesity and type 2 diabetes (Salek *et al.* 2007). Although it has been considered as an inactive biomarker for many years, MNAM has been recently shown to exhibit antidiabetic, antithrombotic, and anti-inflammatory activity (Chlopicki *et al.* 2007, Biedron *et al.* 2008, Mateuszuk *et al.* 2009, Nejabati *et al.* 2018). Chronic MNAM treatment decreases fasting glucose levels and prolongs survival of rats with streptozotocin-induced diabetes where its vasoprotective activity may be involved (Watala *et al.* 2009). Besides, MNAM also improves hepatic insulin sensitivity of mice with type 2 diabetes via activation of SIRT1 and inhibition of forkhead box O1 (FOXO1) acetylation (Zhang *et al.* 2020). Our previous study demonstrated that dietary supplementation with MNAM decreased fasting blood glucose and insulin levels in male mice fed a high-fat diet (HFD) (Hong *et al.* 2015). Notably, Brachs *et al.* recently reported sex-specific differences in body composition, weight, glucose tolerance, and insulin sensitivity in *Nnmt* deficient mice (Brachs *et al.* 2019). Given the previously reported antidiabetic effects of MNAM supplementation in male rodents and the sex-specific metabolic phenotype in *Nnmt* deficient mice, we hypothesized that effects of maternal MNAM treatment in controlling hyperglycemia during pregnancy would be expected.

Therefore, we established a GDM mouse model by feeding C57BL/6N mice with an HFD for 6 weeks before pregnancy and were maintained with HFD until scheduled cesarean delivery. We first aimed to determine whether different doses of MNAM treatment could ameliorate glucose tolerance and insulin tolerance during gestation. The effects of maternal MNAM treatment on the whole body metabolic profile in dams as well as the safety of the fetus were also assessed. We focused on the changes in histology of islet, insulin signaling pathway and nicotinamide adenine dinucleotide (NAD+)/sirtuin system in liver, gonadal white adipose tissue (gWAT), inguinal subcutaneous WAT (sWAT), and skeletal muscle as well as glucose transport in placenta.

## Materials and methods

### Animals and dietary regimen

Eight-week-old female C57BL/6N mice were purchased from Beijing Vital River Lab Pet Technology Co., Ltd. On arrival, mice were randomly group housed and had access to water and a standard CHOW diet (D12450B, Beijing Keao Xieli Feed Co. Ltd., Beijing, China; 11.8% kcal from fat) *ad libitum*. The housing room was maintained on a 12:12 h light–darkness cycle with lights on at 08:00 h. All animal procedures have been approved by the Institutional Animal Care and Use Committee of Xi’an Jiaotong University (XJTU-2022-1184).

After 1 week acclimatization, mice were randomized to receive either CHOW diet (*n* = 40) or an HFD (D12451, Beijing Keao Xieli Feed Co. Ltd., Beijing, China; 45% Kcal from fat; *n* = 60) *ad libitum*. Body weight was recorded weekly. After 6 weeks, mice were mated with C57BL/6N male mice. Two days before mating, 6 h fasting blood glucose (FBG) were determined via a small tail nick using the OneTouch SureStep Test Strips (Johnson & Johnson). Mice were individually housed after mating. Pregnancy was confirmed by the presence of a vaginal plug. The day of vaginal plug expulsion was assigned as GD0.5. Pregnant CHOW-fed mice were randomly remained on CHOW diet (CD, *n* = 7) or CHOW supplemented with 1% MNAM (TCI, Shanghai, China) (CM1%, *n* = 6) throughout gestation. The HFD-fed mice were randomly divided into three subgroups, fed with HFD without or with 0.3% or 1% MNAM (HFD, *n* = 8; HM0.3%, *n* = 7; HM1%; *n* = 8). The concentration of MNAM was chosen based on our previous study and a recent study showing the improvement of 1% MNAM in quantitative insulin sensitivity index (Hong *et al.* 2015) and hepatic insulin sensitivity (Zhang *et al.* 2020). Diet was ground in a blender and powdered MNAM was mixed with the diet to 0.3% v/v (HM0.3%) and 1% v/v (HM1%). Food intake was assessed every 2 days during gestation. Total triglyceride (TG) and cholesterol intake was calculated according to the formula of CHOW diet (TG, 42.6 g/kg; cholesterol, 51.2 mg/kg) and HFD (TG, 236 g/kg; cholesterol, 195.5 mg/kg). Mice with random blood glucose (RGB) ≥ 12 mM tested on 09:00 h of GD15.5 or GD18.5 were diagnosed with GDM in our current study, according the criterion proposed by Li *et al.* (Li *et al.* 2020). Tail blood glucose was measured using a handheld glucose meter (Contour TS, Bayer Diabetes Care).

### Glucose tolerance tests and insulin tolerance tests

Glucose tolerance tests (GTTs) and insulin tolerance tests (ITTs) were performed at GD14.5. In brief, for GTTs, mice were fasted for 6 h (08:00–14:00 h) and then injected intraperitoneally with a 20% saline glucose solution at 2 g/kg body weight. Glucose levels were detected before and 15, 30, 60, 90, and 120 min after injection from a tail blood sample by Contour TS. For the ITTs, mice were fasted for 6 h (08:00–14:00 h) and then injected intraperitoneally with insulin (Humulin U-100) at 0.5 IU/kg body weight. Blood glucose levels were measured before and 15, 30, 60, 90 and 120 min after injection.

### Sample collection

Mice challenged with GTT were fasted for 6 h on GD18.5. The blood was collected by cardiac puncture under isoflurane anesthesia. Following laparotomy, the fetuses, pancreas, bilateral gWAT, bilateral inguinal sWAT, liver, and soleus muscle were carefully removed and weighed. Samples were immediately frozen in liquid nitrogen and then stored at −80°C until further processing, or fixed in 4% paraformaldehyde (PFA) for hematoxylin–eosin (H&E) staining. Litter size and weight of each fetus in each litter were recorded after cesarean delivery. Mice challenged with ITT were fasted for 6 h on GD15.5 and injected with saline or insulin (Humulin U-100) at 0.5 IU/kg body weight. After 10 min, blood and tissues were collected as per protocols at GD18.5.

### Measurements of plasma metabolic profile

On day of sacrifice, RGB and 6-h FBG levels were determined from a tail blood sample by Contour TS. Plasma samples collected on the day of sacrifice were used for the following assays: plasma triglyceride (TG), total cholesterol levels (TC), glutamic-pyruvic transaminase (GPT) and glutamic-oxaloacetic transaminase (GOT) levels were detected using commercial kits (Nanjing Jiancheng Bioengineering Institute, Nanjing, China); plasma insulin levels were measured using an ELISA kit (Cusabio Biotech CO., LTD, Wuhan, China).

Six-hour fasting blood from nonpregnant mice was collected by cardiac puncture under isoflurane anesthesia. Plasma MNAM levels were determined using LC-MS strategy, which was performed on a Shimadzu NexeraLC-30AD UHPLC system with a Waters Acquity UPLC HSS T3 column (1.7 μm, 2.1 mm × 100 mm) and an AB SCIEX QTRAP 5500 mass spectrometer. Concentrations were quantitated based on the peak area compared to a standard curve. Body fat mass was determined by an MRI analyzer (Spinsci Solutions Ptd. Ltd, Singapore).

### Histological analysis

The pancreas tail was cut and fixed with 4% paraformaldehyde (PFA). Five sections of each pancreas (paraffin embedded) were sliced at 5 μm thickness, separated by at least 200 μm, and stained with H&E. For visualization, a light microscope (BX53; Olympus) was used. Data were collected from five mice in each group, at 400× magnification. All islets composed of more than 10 cells were marked. Total islet number, islet size (μm^2^) and total pancreas areas (μm^2^) in each section were counted or measured using ImageJ software (National Institutes of Health). Islet density was calculated by the total number of islets divided by total area of pancreas in each section.

H&E and Oil Red O staining in liver samples were prepared as described previously (Wei *et al.* 2023). Placentae were fixed in 4% PFA, embedded in paraffin, and sectioned in the vertical plane at 3 μm thickness. GLUT1 immunohistochemistry in placenta was performed using a monoclonal anti-GLUT1 antibody (Proteintech, Wuhan, China) (66290-1-Ig, 1:500). The sections were visualized under a microscopy (BX53; Olympus).

### RNA extraction, cDNA synthesis, and quantitative polymerase chain reaction

Total RNA was extracted by isolation kit (R0027, Beyotime, Beijing, China) according to the manufacturer’s instructions. cDNA was prepared with Reverse Transcription Kit (K1622, ThermoFisher Scientific). Gene expression was quantified by qPCR using SYBR Green Pro Taq HS (AG11701, Accurate Biology, Changsha, Hunan, China) in a iQ5 PCR thermal cycler (Bio-Rad). Relative gene expression was calculated by geometric averaging of multiple internal control genes, *Gapdh*, *Cyclophilin*, and *Actb* (Vandesompele *et al.* 2002). Primer sequences are available upon request.

### Western blotting analysis

Tissues were homogenized in RIPA buffer (Beyotime, China) and lysed for 30 min at 4°C. Protein concentration was measured by the Bradford assay (Bio-Rad). Twenty micrograms of protein were separated using 10% TGX stain-free acrylamide gels (Bio-Rad), then transferred to polyvinylidene difluoride membranes (Millipore). The membranes were blocked and then incubated with the following primary antibodies overnight at 4°C: total Akt (#9272) and phosphorylated Akt (#4060) (Cell Signaling Technology). Signals were detected using ChemiDoc Touch Imaging System (Bio-Rad). Densitometry analysis was performed with Image Lab software (Bio-Rad). Normalization was carried out with reference to GAPDH (Bioss, Beijing, China) or the total lane protein, which was detected by imaging in stain-free gels.

Plasma membrane (PM) fractions of skeletal muscles and sWAT were fractioned using the Surface and Cytoplasmic Protein Reagent Kit (Cat#P0033; Beyotime, Shanghai, China) according to the manufacturer’s instructions and subjected to Western blotting analysis for GLUT4 (66846-1-Ig, Proteintech, China). PM protein was normalized to ATPase Na+/K+ transporting subunit alpha 1 (APT1A1) (bs-42166R, Bioss), and total protein was normalized to GAPDH. The ratio of PM to total GLUT4 protein was quantified.

### Statistical analysis

Data are presented as mean ± s.e.m. Pairwise differences were tested by Student’s *t*-test. One-way ANOVA or repeated two-way ANOVA followed by Tukey’s *post hoc* tests was used to compare differences among multiple groups. Pearson’s correlation was employed to assess the association. Significance was set at *P* < 0.05. All outliers have been included in statistical analyses.

## Results

### Maternal MNAM treatment impairs glucose tolerance at GD14.5

**Figure 1:**
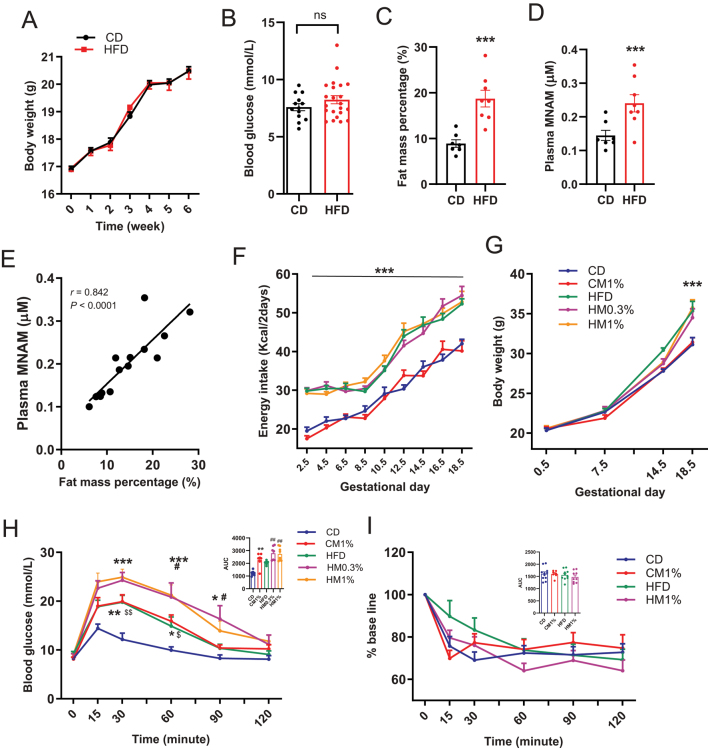
Maternal MNAM treatment impairs glucose tolerance. (A) Body weight during 6-week HFD challenge before gestation (CD, *n* = 40; HFD, *n* = 60). (B) Six-hour fasting blood glucose before mating. (C, D) Body fat mass percentage and plasma MNAM levels in nonpregnant mice challenged with 6 weeks CHOW or HFD (CD, *n* = 7; HFD, *n* = 8). (E) Correlation between fat mass percentage and plasma MNAM levels (*n* = 15). (F, G) Body weight and food intake during gestation. (H) GTT at GD14.5 and area under the curve (AUC) (CD, *n* = 7; CM1%, *n* = 6; HFD, *n* = 8; HM0.3%, *n* = 7; HM1%; *n* = 8 for B–F). (I) ITT at GD14.5 and AUC (CD, *n* = 11; CM1%, *n* = 7; HFD, *n* = 10; HM1%; *n* = 9). Data are presented as mean ± s.e.m. Statistical significance was tested by Student’s *t*-test (B ,C, D), repeated two-way (A, F, G, H, I) or one-way ANOVA (AUC in H, I) followed by Tukey’s *post hoc* tests. Pearson’s correlation was employed to assess the association (E). ‘ns’ is not significant, *P < 0.05, **P < 0.01, ***P < 0.001 versus CD group; #*P* < 0.05 versus HFD group, $*P* < 0.05, CM1% versus CD. A full color version of this figure is available at https://doi.org/10.1530/JME-23-0126.

Six weeks HFD feeding before mating did not change the body weight or FGB levels (Fig. 1A and B) but significantly increased the fat mass percentage compared with CHOW diet feeding (Fig. 1C). Plasma MNAM levels of HFD-fed mice prior to pregnancy were significantly higher than control mice (Fig. 1D), and positively correlated with fat mass percentage (r = 0.842, *P* < 0.0001, Fig. 1E). Although the energy intake of the HFD groups were higher than that of the CHOW group throughout pregnancy, the higher body weight in HFD groups were only observed at GD18.5 (Fig. 1F and G). However, MNAM supplementation either in CHOW diet or HFD had no significant effect on dam’s body weight or energy intake compared with CD and HFD group, respectively (Fig. 1F and G). Glucose tolerance at GD14.5 was markedly impaired by HFD feeding, with higher blood glucose at 30 and 60 min and a higher glucose area under curve (AUC) compared to CD group (Fig. 1H). Contrary to our expectation, MNAM supplementation in either CHOW diet or HFD impaired glucose tolerance. CM1%, HM0.3%, and HM1% mice all showed higher blood glucose levels and higher glucose AUC compared with CD group and HFD group (Fig. 1H). No differences in blood glucose or AUC were presented between 0.3% and 1% MNAM treatment (Fig. 1H). ITT at GD14.5 was not affected by either HFD feeding or MNAM treatment, with equal reduction from baseline at every time point and comparable AUC among groups (Fig. 1I). Glucose tolerance was not changed in nonpregnant mice challenged with 6 weeks of HFD consumption and 2 weeks of MNAM treatment (Supplementary Fig. 1, see the section on supplementary materials given at the end of this article).

### Maternal MNAM treatment ameliorates HFD-induced adipose tissue mass gain

**Figure 2:**
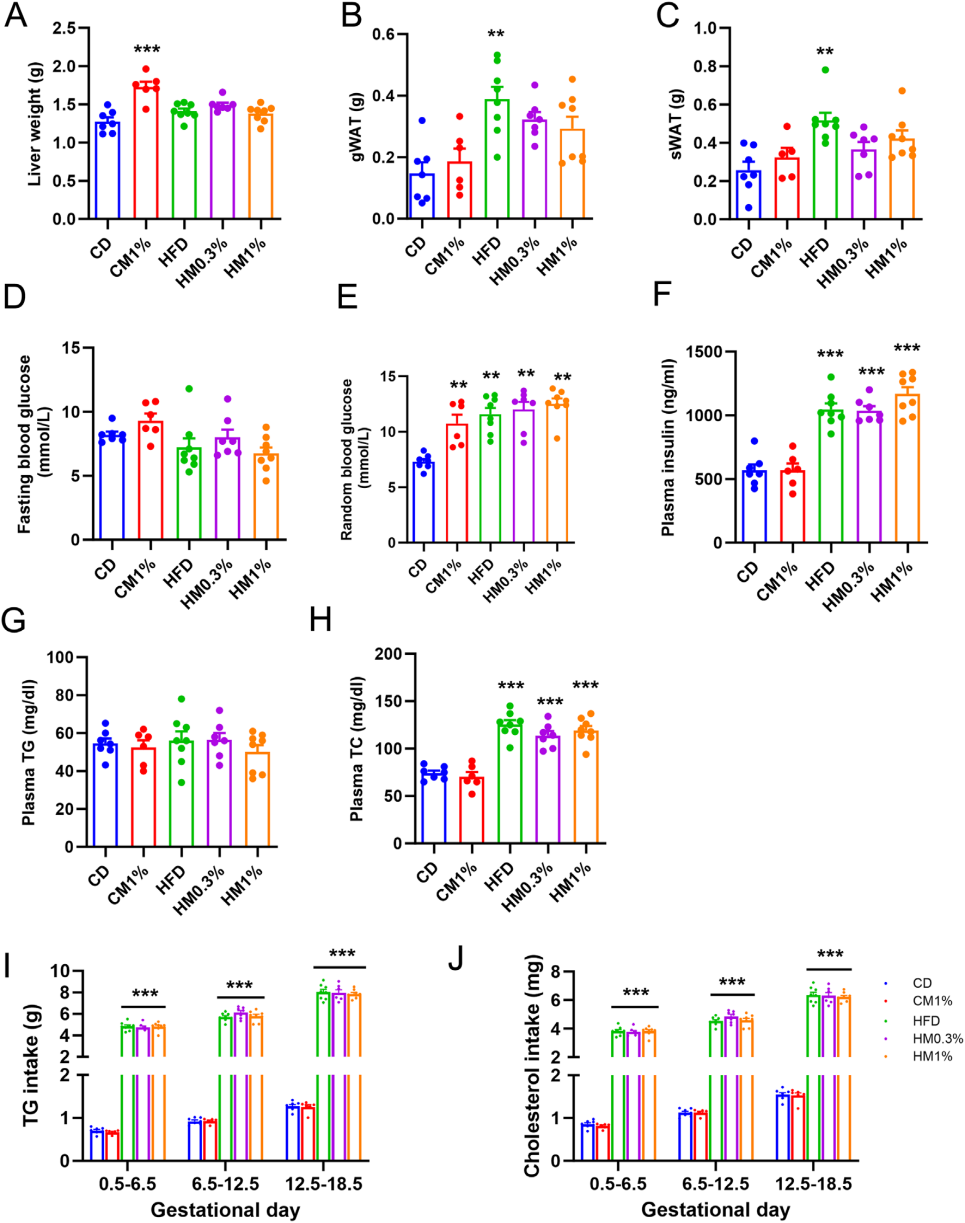
Maternal MNAM treatment ameliorates HFD-induced adipose tissue mass gain. (A, B, C) Weights of liver, bilateral gonadal white adipose tissue (gWAT), and inguinal subcutaneous WAT (sWAT) at day of sacrifice (GD18.5). (D, E, F, G, H) 6-h fasting blood glucose, random blood glucose, plasma insulin, plasma triglycerides, and plasma total cholesterol levels at the GD18.5. (I, J) Total triglyceride and cholesterol intake during early (GD0.5–GD6.5), middle (GD6.5–GD12.5), and late pregnancy (GD12.5–GD18.5). Data are presented as mean ± s.e.m. CD, *n* = 7; CM1%, *n* = 6; HFD, *n* = 8; HM0.3%, *n* = 7; HM1%; *n* = 8. Statistical significance was tested by one-way ANOVA followed by Tukey’s *post hoc* tests. *P < 0.05, **P < 0.01, ***P < 0.001. A full color version of this figure is available at https://doi.org/10.1530/JME-23-0126.

At the end of experiment, we evaluated the effect of MNAM treatment on whole-body metabolic profile as well as the safety of the fetus. CM1% group mice showed significantly higher liver weight than CD group, while no differences in liver weight were detected among three HFD-fed subgroups (Fig. 2A). Compared with CD group, more gWAT and sWAT mass were observed in HFD groups. 1% MNAM treatment in HFD restored the gWAT and sWAT mass (Fig. 2B and C). There were no differences in 6-h FBG among five groups (Fig. 2D), but higher RBG levels were observed in CM1% group mice and three HFD-fed mice groups compared with CD group (Fig. 2E). HFD, HM0.3%, and HM1% groups all displayed elevated 6-h fasting insulin (FIN) (Fig. 2F). No significant differences in plasma TG were seen among five groups (Fig. 2G). HFD feeding significantly increased the plasma total cholesterol (TC) levels, while MNAM treatment had no effect on TC levels (Fig. 2H). TG and cholesterol intake was calculated during early-, middle-, and late-pregnancy periods. As shown in Fig. 2I and J, consumption of TG and cholesterol were both significantly higher in HFD, HM0.3%, and HM1% groups than CHOW group, suggesting that higher cholesterol intake contributed to increased plasma TC levels.

### Maternal MNAM treatment does not alter pancreatic morphology or insulin signaling but reduces GLUT4 in skeletal muscle

**Figure 3:**
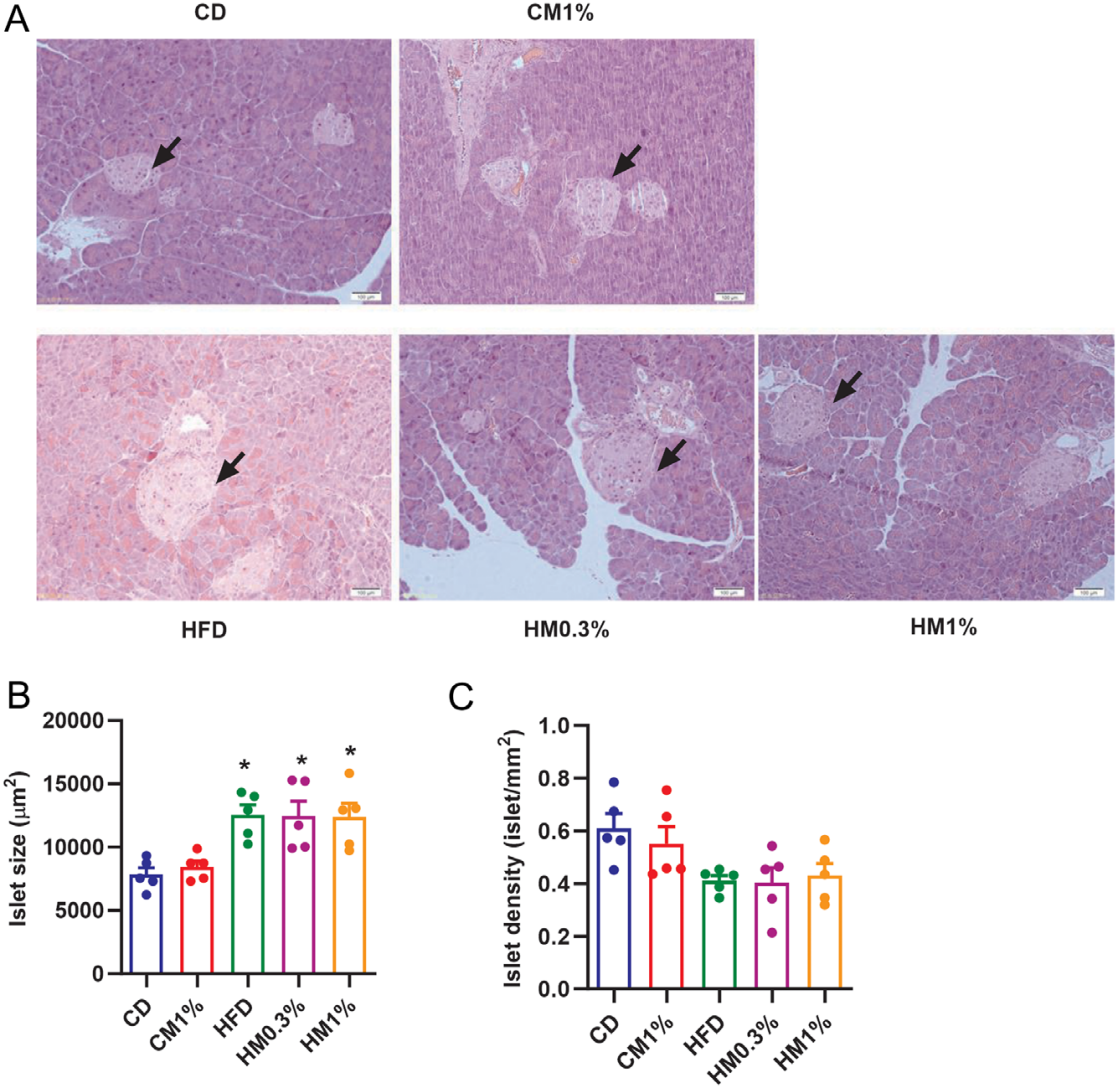
Maternal MNAM treatment does not alter pancreatic morphology. (A) Representative photomicrographs of pancreas sections subjected to H&E staining. (B) Islet size. (C) Islet density. Data are presented as mean ± s.e.m. Statistical significance was tested by one-way ANOVA followed by Tukey’s *post hoc* tests. *n* = 5/group. *P < 0.05. Magnification: 400×, scale bar: 100 μm. A full color version of this figure is available at https://doi.org/10.1530/JME-23-0126.

To explore the reasons for impaired glucose tolerance, we examined whether MNAM treatment induced structural changes in pancreatic islets and the insulin signaling pathway in liver, WAT and skeletal muscle. As shown in Fig. 3A, B, and C, islet sizes in three HFD-fed subgroups were all larger than CD group. However, islet density did not differ among five groups. MNAM treatments had no effect on either islet size or islet density.

**Figure 4:**
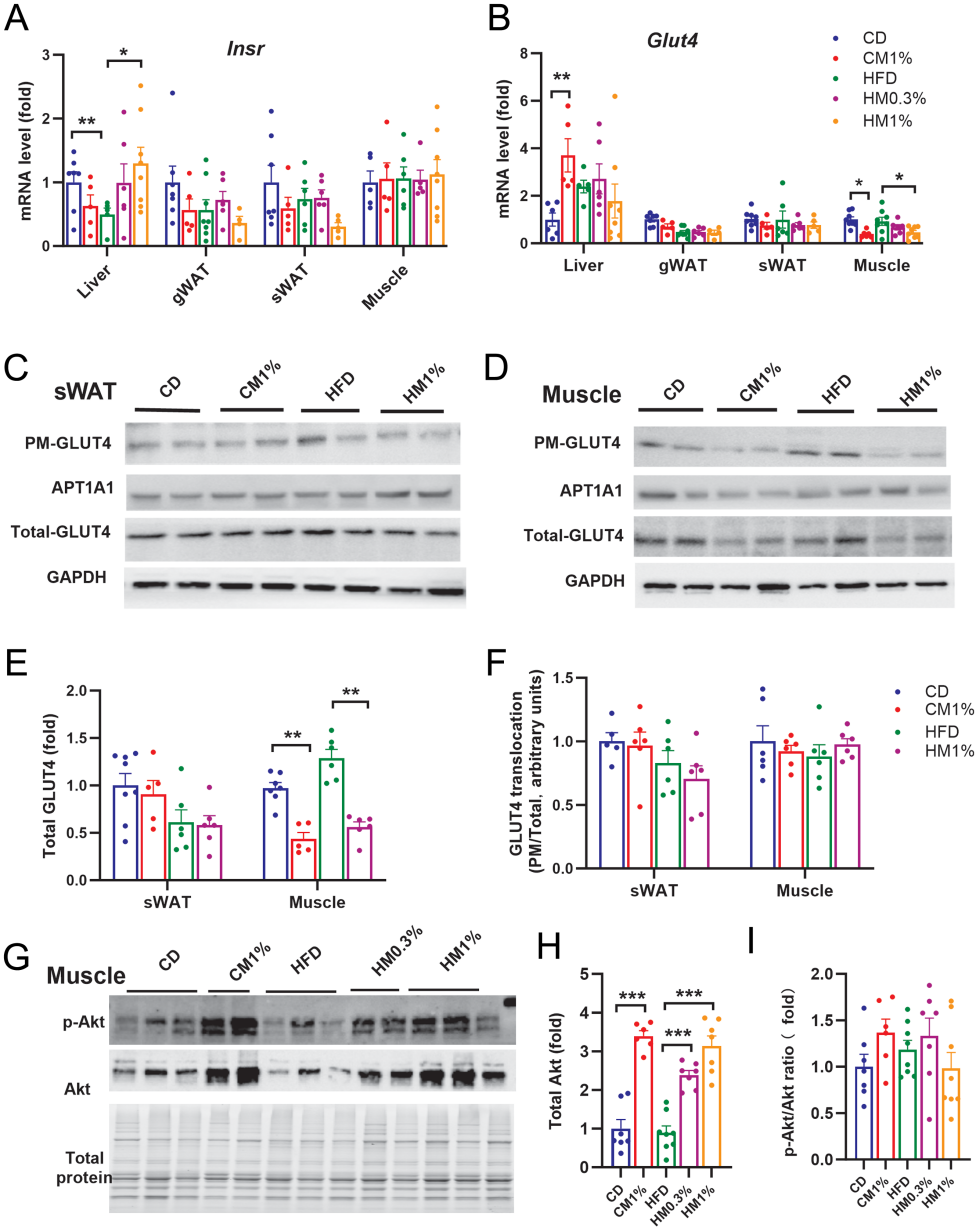
Maternal MNAM treatment does not alter insulin signaling but reduces GLUT4 in skeletal muscle. (A, B) mRNA levels of insulin receptor (*Insr*) and glucose transporter 4 (*Glut4*) in liver, gWAT, sWAT, and skeletal muscle. (C, D) Representative Western blots of GLUT4 protein in plasma membrane (PM) and total tissue lysate in sWAT and skeletal muscle. (E) Quantification of total GLUT4 in sWAT and skeletal muscle. (F) Ratio of PM to total GLUT4 protein. PM protein was normalized to APT1A1, and total protein was normalized to GAPDH. (G) Representative Western blots of total and phosphorylated Akt (S473) in skeletal muscle. (H, I) Quantification of total Akt and p-Akt/Akt ratio in skeletal muscle. Data are presented as mean ± s.e.m. *n* = 6–8/group. Statistical significance was tested by one-way ANOVA followed by Tukey’s *post hoc* tests. *P < 0.05, **P < 0.01, ***P < 0.001. A full color version of this figure is available at https://doi.org/10.1530/JME-23-0126.

Hepatic *Insr* gene expression was significantly decreased by HFD feeding and was restored by 1% MNAM supplementation in HFD (Fig. 4A). *Glut4* gene expression in liver was significantly increased in CM1% group compared with CD group, but was not changed by MNAM treatments in HFD-fed groups. In contrast, *Glut4* gene expression in skeletal muscle was significantly decreased in both CM1% and HM1% group compared with CD and HFD group respectively (Fig. 4B). No differences in either *Insr* or *Glut4* gene expression in gWAT or sWAT were detected among five groups (Fig. 4A and B). Total GLUT4 protein was lowered by MNAM in skeletal muscle, while the ratio of PM-GLUT4 to total-GLUT4 was not changed by HFD feeding or MNAM treatment in either sWAT or skeletal muscle (Fig. 4C, D, E, and F). Total Akt protein expression in skeletal muscle was significantly increased by MNAM treatment in both CHOW and HFD (Fig. 4G and H), while no changes of p-Akt-to-Akt ratios among groups were detected (Fig. 4I). Expression of phosphorylated Akt or total Akt was not affected by MNAM in either liver (Supplementary Fig. 2A, B, and C) or sWAT (Supplementary Fig. 2D, E, and F). Unchanged ITT, GLUT4 translocation as well as the phosphorylation of AKT in liver, sWAT, or skeletal muscle indicate that insulin sensitivity was not changed by either HFD feeding or MNAM treatment. However, the decreased total GLUT4 in skeletal muscle induced by MNAM may contribute to impaired GTT.

### Maternal MNAM treatment dampens NAD+ salvage synthesis in skeletal muscle

**Figure 5:**
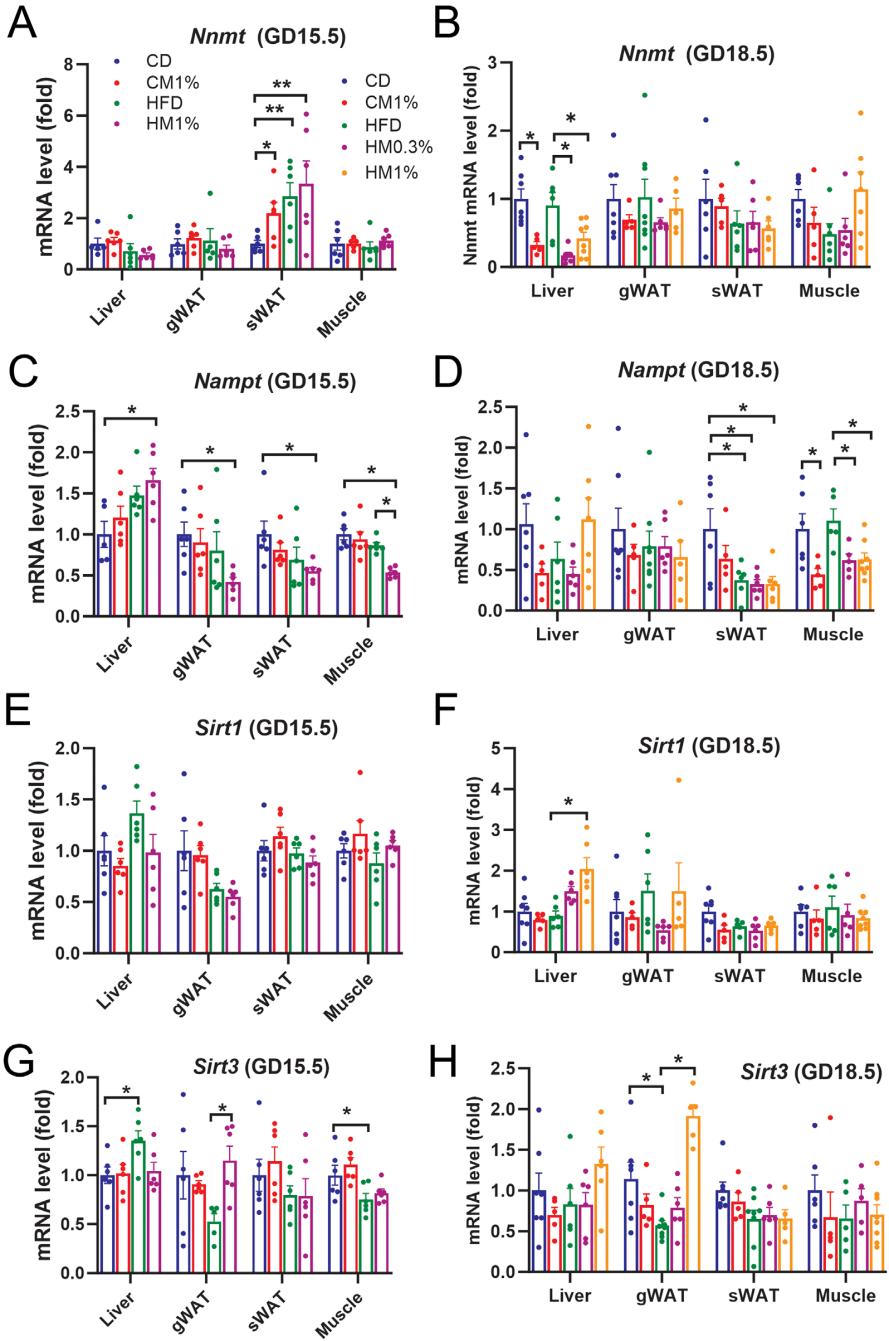
Maternal MNAM treatment dampens NAD+ salvage synthesis in skeletal muscle. Relative mRNA expression of key enzymes in NAD+ metabolism, nicotinamide *N*-methyltransferase (*Nnmt*) at GD15.5 and GD18.5 (A, B), nicotinamide phosphoribosyltransferase (*Nampt*) at GD15.5 and GD18.5 (C, D), *Sirt1* at GD15.5 and GD18.5 (E, F), *Sirt3* at GD15.5 and GD18.5 (G, H ) in liver, gWAT, sWAT, and skeletal muscle. Data are presented as mean ± s.e.m. *n* = 5–/group. Statistical significance was tested by one-way ANOVA followed by Tukey’s *post hoc* tests. *P < 0.05, **P < 0.01. A full color version of this figure is available at https://doi.org/10.1530/JME-23-0126.

Since MNAM is the major metabolite of nicotinamide via NNMT, which is shown to regulate energy expenditure partly through increased NAD+ content (Kraus *et al.* 2014, Komatsu *et al.* 2018), we were curious whether NAD+ metabolism was changed by MNAM treatment. mRNA expression of *Nnmt* and key genes in NAD+ salvage synthesis pathway, nicotinamide phosphoribosyltransferase (*Nampt*), and NAD+ consumers, *Sirt1* and *Sirt3* were analyzed. *Nnmt* gene expression at GD15.5 was increased in sWAT of CM1%, HFD, and HM1% group compared with CD group (Fig. 5A), while hepatic expression at GD18.5 was significantly reduced in CM1%, HM0.3%, and HM1% groups compared with CD group and HFD group respectively (Fig. 5B). *Nampt* gene expression at GD15.5 was significantly increased in liver of HM1% group but decreased in gWAT, sWAT, and skeletal muscle of HM1% group compared with CD group (Fig. 5C), while expression at GD18.5 was further reduced in the sWAT of mice in three HFD-fed subgroups compared with CD group (Fig. 5D). Notably, *Nampt* gene expression at GD18.5 in skeletal muscle was significantly reduced by MNAM treatment in both CHOW- and HFD-fed mice (Fig. 5D). Gene expression of *Sirt1* in liver at GD18.5 was significantly increased in HM1% group compared with HFD group, while no differences at GD15.5 were found in any tissues (Fig. 5E and F). *Sirt3* gene expression was increased in gWAT of HM1% group at GD15.5 and GD18.5 compared with HFD group (Fig. 5G and H). The expression of housekeeping genes *Gapdh*, *Cyclophilin* and *Actb* among groups did not change (Supplementary Fig. 3). Collectively, decreased *Nampt* in skeletal muscle is a relatively stable effect of MNAM treatment at both mid-gestation and late gestation.

### Maternal MNAM treatment alleviates HFD-induced fatty liver while boosting gluconeogenesis

**Figure 6:**
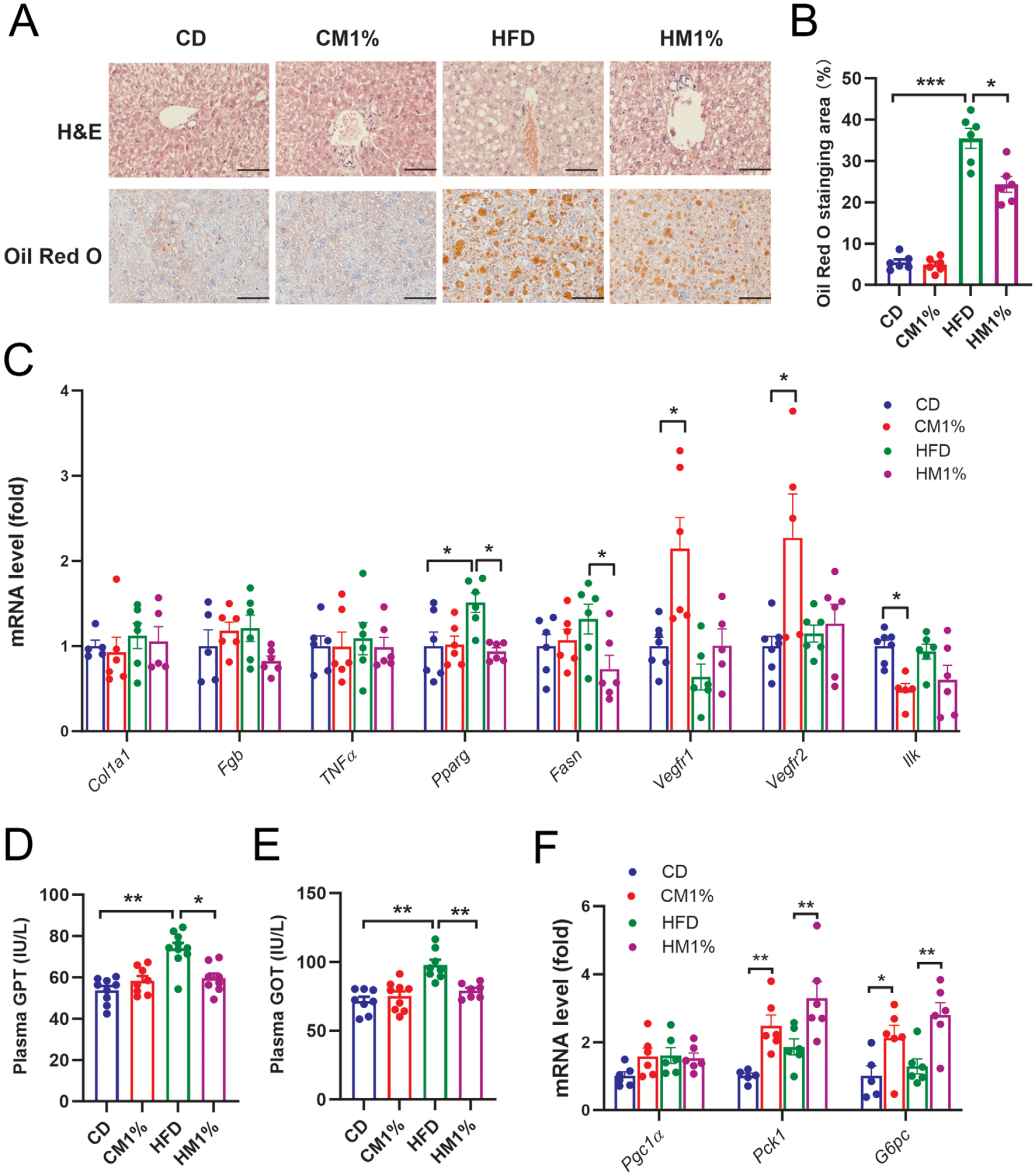
Maternal MNAM treatment alleviates HFD-induced fatty liver while boosting gluconeogenesis. (A) Histological analysis of liver sections, H&E staining periportal area and Oil Red O staining (magnification: 400×, scale bar: 100 μm). (B) Quantification of Oil Red O staining area of hepatic sections (*n* = 6/group). (C) Hepatic mRNA expression of genes related to fibrosis and inflammation (*Colla1*, *Fgb*, *Tnfα*), lipogenesis (*Pparg*, *Fasn*) and proliferation (*Vegfr1*, *Vegfr2*, *Ilk*) (*n* = 6/group). (D, E) Plasma glutamic-pyruvic transaminase (GPT) and glutamic-oxaloacetic transaminase (GOT) levels at GD15.5 (*n* = 8–10/group). (F) Hepatic mRNA levels of key genes in gluconeogenesis, *Pgc1α*, *Pck1*, and* G6pc* (*n* = 6–7/group). Data are presented as mean ± s.e.m. Statistical significance was tested by one-way ANOVA followed by Tukey’s *post hoc* tests. *P < 0.05, **P < 0.01, ***P < 0.001. A full color version of this figure is available at https://doi.org/10.1530/JME-23-0126.

The observation of increased liver weight in CM1% group led us to test whether elevated lipid storage, proliferation or fibrosis occurred. To evaluate hepatic lipid accumulation, we performed Oil Red O staining accompanied with H&E staining. Hepatic lipid content was significantly increased in HFD group, which was lowered by MNAM treatment evidenced by the higher the Oil Red O staining area (Fig. 6A and B). Consistently, MNAM treatment reversed elevated mRNA levels of the major lipogenic genes, *Pparg* and *Fasn,* in HFD group (Fig. 6C), as well as increased plasma levels of GPT and GOT (Fig. 6D and E). No differences in expression of fibrosis related genes, collagen type I alpha 1* (Col1a1)*, fibrinogen (*Fgb)*, or *Tnfα* were found among groups (Fig. 6C). Interestingly, expression of genes involved in hepatocyte proliferation during chronic injury, vascular endothelial growth factor receptor 1 and 2 (*Vegfr1*, *Vegfr2*) were significantly elevated in CM1% group compared with CD group, while gene expression of integrin linked kinase (*Ilk*), a vital regulator in remodeling of hepatic matrix, was decreased by MNAM (Fig. 6C). CM1% and HM1% mice showed increased hepatic phosphoenolpyruvate carboxykinase 1 (*Pck1*) and glucose-6-phosphatase catalytic (*G6pc*) gene expression compared with CD and HFD group respectively (Fig. 6F), indicating that hepatic gluconeogenesis was promoted by MNAM treatment.

### Maternal MNAM treatment impairs antioxidant defenses in skeletal muscle but reduces inflammation in WAT

**Figure 7:**
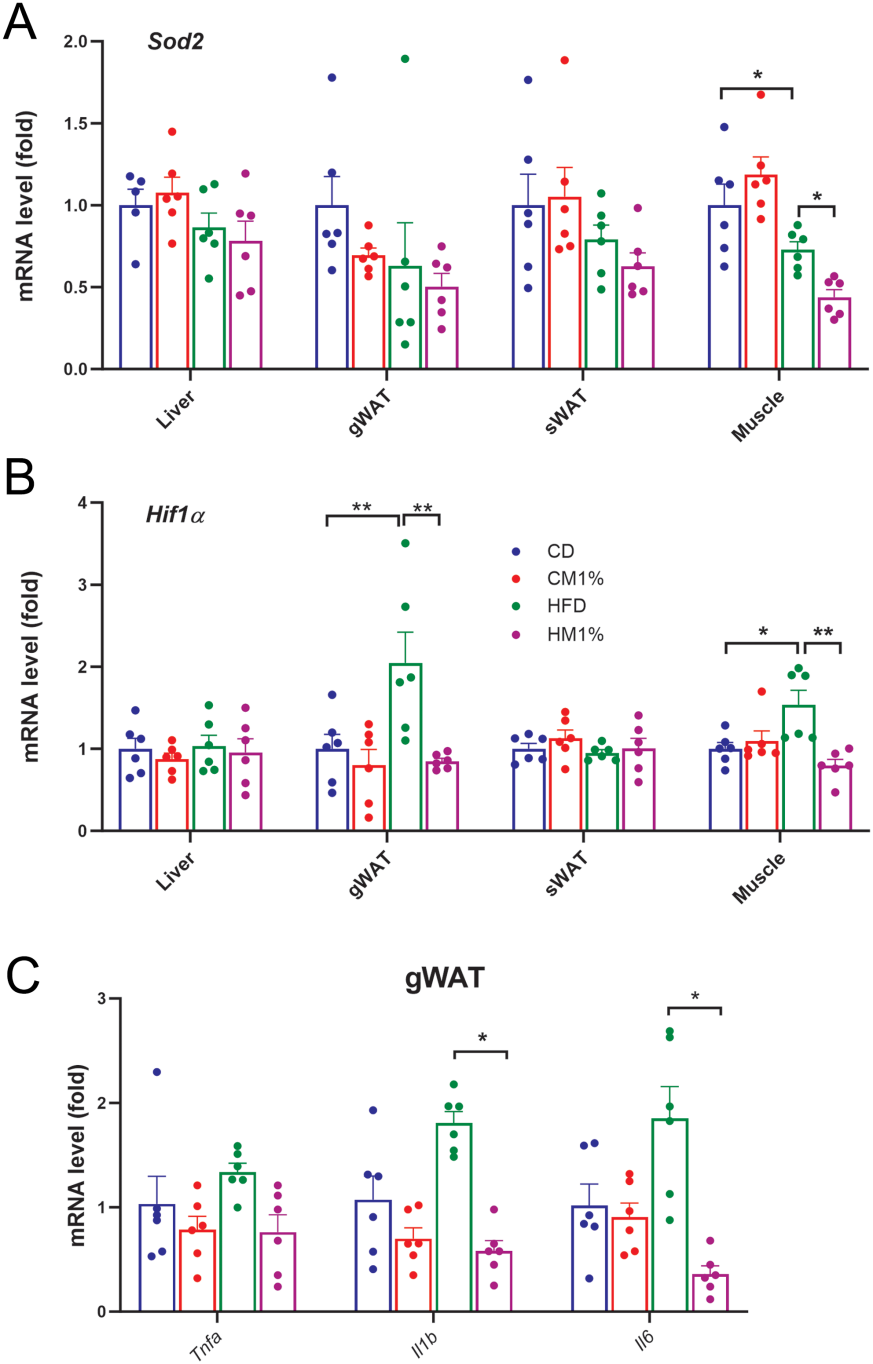
Maternal MNAM treatment impairs antioxidant defenses in skeletal muscle but reduces inflammation in WAT. (A, B) Relative mRNA levels of *Sod2* and *Hif1a* in liver, gWAT, sWAT, and skeletal muscle collected at GD15.5 (*n* = 6/group). (C) Relative mRNA levels of inflammatory genes (*Tnfα*, *Il1b*, *Il6*) in gWAT (*n* = 6/group). Data are presented as mean ± s.e.m. Statistical significance was tested by one-way ANOVA followed by Tukey’s *post hoc* tests. * *P* < 0.05, **P < 0.01. A full color version of this figure is available at https://doi.org/10.1530/JME-23-0126.

Glucose intolerance is frequently associated with increase of reactive oxygen species (ROS) and oxidative stress (Zhuang *et al.* 2021). Gene expression of superoxide dismutase 2 (*Sod2*) is significantly decreased in the skeletal muscle of HFD group than in CD group mice, which was further decreased in HM1% group compared with HFD group (Fig. 7A). HFD feeding significantly increased the gene expression of hypoxia-inducible factor 1α (Hif1α) in gWAT and skeletal muscle, which were reversed by MNAM treatment (Fig. 7B). MNAM treatment significantly reduced the gene expression of* Il1b* and *Il6* in gWAT compared with HFD group (Fig. 7C), while these inflammatory genes were not altered by MNAM in sWAT (Supplementary Fig. 4). MNAM was previously shown to stimulate lipolysis in mature rat adipocytes (Strom *et al.* 2018). However, gene expression of *Atgl* and *Hsl* were equal among five groups (Supplementary Fig. 5).

### Maternal MNAM treatment diminishes glucose transport in placenta

**Figure 8:**
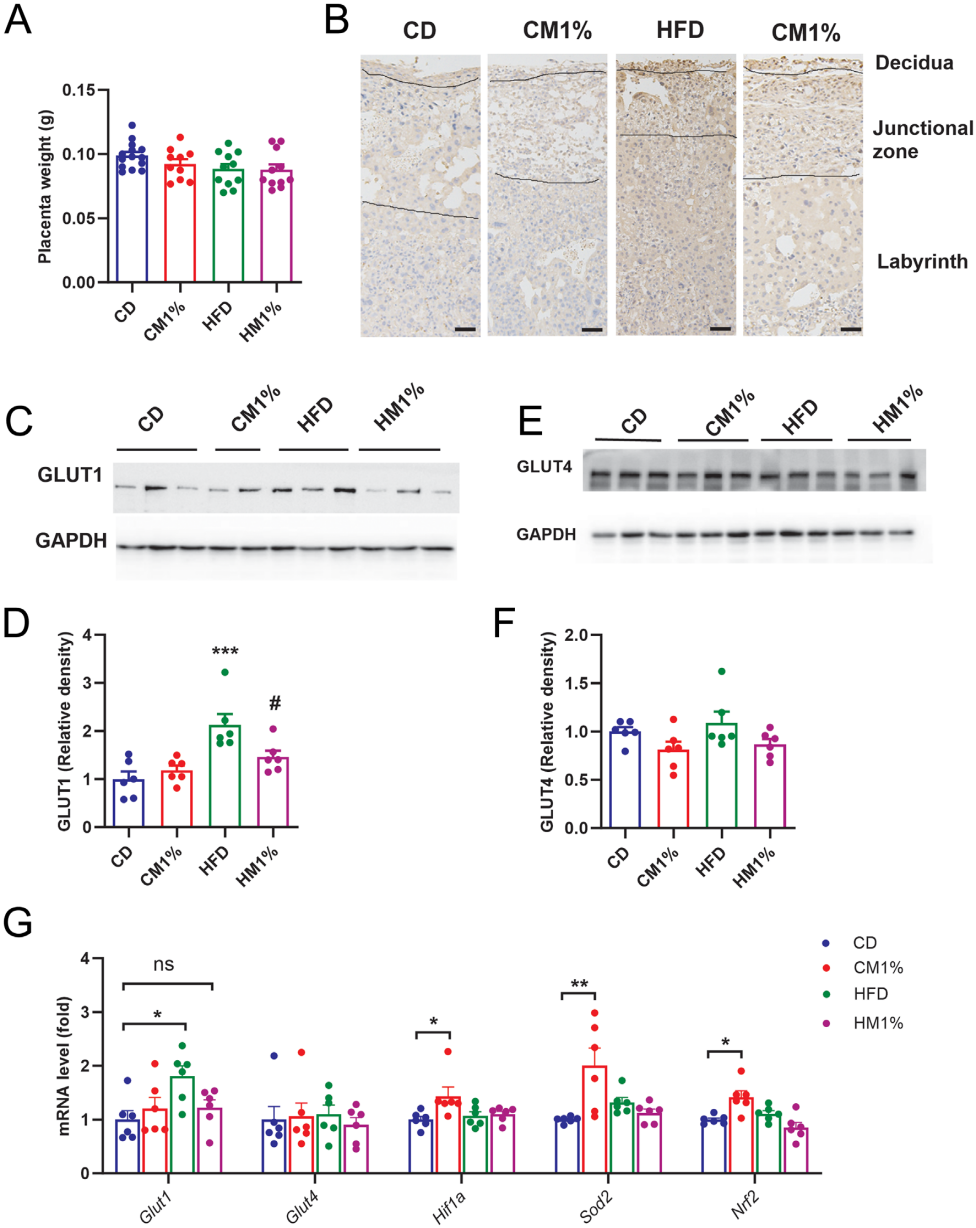
Maternal MNAM treatment diminishes glucose transport in placenta. (A) Weight of placenta collected at GD15.5. (*n* = 10–14/group). (B) Immunohistochemical staining of GLUT1 in placenta collected at GD15.5. (magnification: 200×, scale bar: 200 μm). (C, D) Representative Western blots of GLUT1 and GLUT4 in placenta collected at GD15.5 (E, F) quantification of Western blotting analysis. (G) mRNA expression of glucose transport related gene (*Glut1*, *Glut4*) and oxidative stress related genes (*Hif1a*, *Sod2*, *Nrf2*). Data are presented as mean ± s.e.m. *n* = 6/group. Statistical significance was tested by one-way ANOVA followed by Tukey’s* post hoc* tests. ‘ns’ is not significant, *P < 0.05, **P < 0.01. A full color version of this figure is available at https://doi.org/10.1530/JME-23-0126.

Placenta weight and glucose transporters were next evaluated. Placenta weight did not differ among five groups at GD15.5 (Fig. 8A). Immunohistochemical analysis showed higher expression of GLUT1 protein in HFD group but not in HM1% group compared with CD group (Fig. 8B). Consistently, placental gene and protein expression of GLUT1 were significantly increased by HFD consumption, while MNAM treatment normalized it (Fig. 8C, D, and G). No significant differences in GLUT4 gene and protein content were found among groups (Fig. 8E, F, and G). However, the litter size and fetus weight were equal among groups (Supplementary Fig. 6).

## Discussion

NNMT has recently emerged as a critical player in the regulation of energy homeostasis through targeting NAD+ metabolism (Kraus *et al.* 2014, Pissios 2017, Komatsu *et al.* 2018, Brachs *et al.* 2019, Song *et al.* 2020). Our previous study showed that NNMT regulates hepatic gluconeogenesis and cholesterol metabolism via MNAM-mediated Sirt1 protein stabilization (Hong *et al.* 2015). Several studies reported the antidiabetic effect of MNAM supplementation in type 1 diabetic rats and obese type 2 diabetes mellitus mice (Watala *et al.* 2009, Zhang *et al.* 2020). However, evidence of MNAM treatment managing glucose homeostasis during gestation is limited. To our surprise, MNAM treatment did not improve but impaired GTT without influence on ITT at mid-pregnancy. Impaired glucose tolerance was not only observed in HM1% group but also in the CM1% group, which showed similar glucose tolerance as the HFD group. During normal pregnancy, β-cells undergo hyperplasia in order to meet the metabolic demands of pregnancy, while during gestational diabetes, β-cells fail to compensate for the demands of pregnancy on a background chronic insulin resistance (Plows *et al.* 2018). In the study, HFD increased islet size and FIN levels, while neither was changed by MNAM treatment. In line with the unchanged ITT, phosphorylation of AKT and GLUT4 translocation did not differ in either sWAT or skeletal muscle among groups. However, we found that MNAM decreased gene and protein expression of GLUT4 in skeletal muscle. In short, insulin signaling was not influenced in our mice model, and decreased GLUT4 in skeletal muscle may contribute to impaired GTT.

Zhang *et al.* recently demonstrated that MNAM reduced FBG and improved hepatic insulin sensitivity in male mice with type 2 diabetes (Zhang *et al.* 2020). In contrast, FBG or insulin sensitivity was not affected by maternal MNAM treatment in our study. The discrepancy may result from the different animal model in Zhang's study: *ob/ob* male mice treated with MNAM for 8 weeks. Brachs *et al.* recently reported that *Nnmt* deficiency enhances insulin sensitivity in HFD-fed males and improves body composition in Western diet-fed females without affecting glucose tolerance in males or females, suggesting a sex-specific effect of *Nnmt* deletion (Brachs *et al.* 2019). Our data showed that MNAM levels in nonpregnant female mice positively correlated with percentage body fat, which is consistent with increased urinary MNAM in male obese mice (Salek *et al.* 2007). This finding suggests that differences in intrinsic MNAM is not the reason for a sex-specific effect of MNAM.

To explore the mechanisms involved, the NAD+/sirtuin system was evaluated in major metabolic tissue, liver, gWAT, sWAT, and skeletal muscle. Expression of key enzymes in NAD+/sirtuin showed different patterns across tissues and throughout pregnancy. *Nnmt* gene expression was suppressed in liver by MNAM at GD18.5 but increased in sWAT by MNAM at GD15.5. Our previous study showed that HFD consumption increases NNMT expression in sWAT but not in liver (Wei *et al.* 2020, 2023). Our data further indicate the tissue-specific function of Nnmt. Nnmt knockdown improves glucose tolerance and protects against diet-induced obesity (Kraus *et al.* 2014). Therefore, elevated NNMT in sWAT may contribute to impaired GTT at mid-pregnancy. As a key enzyme in NAD+ biosynthesis, *Nampt* gene expression was decreased in gWAT, sWAT, and skeletal muscle of HM1% mice, but increased in livers of HM1% mice, suggesting a negative regulation of Nampt in WAT and skeletal muscle but positive in liver by MNAM. Hepatic Sirt1 at GD18.5 was also enhanced in HM1%, consistent with our previous study showing positive regulation of sirt1 by MNAM (Hong *et al.* 2015). NAMPT-mediated NAD+ biosynthesis suppresses activation of hepatic stellate cells and protects against liver fibrosis in mice (Xu *et al.* 2021). Thus, elevated hepatic *Nampt* and *Sirt1* together with decreased *Nnmt* suggest improved hepatic NAD+/sirtuins by MNAM.

Substantial studies have demonstrated the advantages of boosting NAD+ and hepatocyte-specific *Nnmt* deletion in liver diseases (Song *et al.* 2020, Mukherjee *et al.* 2021, Li *et al.* 2022). Although no inflammatory genes were changed, MNAM reduced lipid accumulation and ameliorated hepatic function, which further confirmed improved hepatic NAD+ metabolism. Expression of genes related to gluconeogenesis, *Pck1* and *G6p*, were also upregulated in liver by MNAM, which is in line with our previous study, which demonstrated that NNMT is a positive regulator of hepatic gluconeogenesis through Sirt1 protein stabilization by MNAM (Hong *et al.* 2015). In individuals with compromised insulin signaling, insulin fails to suppress hepatic gluconeogenesis, even in the fed state (Hatting *et al.* 2018). Thus, though increased *Insr* gene expression was seen in the HM1% group, hepatic gluconeogenesis is still increased by MNAM, which may be responsible for higher blood glucose after glucose injection. The NNMT–MNAM axis induces lipolysis in adipose tissues and gluconeogenesis in livers during fasting to provide energy for muscles (Strom *et al.* 2018, Nejabati *et al.* 2022). However, in our mice model, MNAM treatment did not change lipolysis in adipose tissue, suggesting that MNAM is not sufficient to induce lipolysis *in vivo* during gestation. Analyses of lipid storage, fibrosis, and proliferation indicate that hepatocyte proliferation in CM1% group contributes to the increased liver weight evidenced by higher expression of *Vegfr1* and *Vegfr2.* Moreover, several studies have also revealed the protective effects of MNAM supplementation in various liver injury models (Sternak *et al.* 2010, Ding *et al.* 2021). Additional studies are required to identify the mechanisms involved in increased hepatocyte proliferation.

In skeletal muscle, NAMPT is induced by energy deprivation through an AMPK-dependent mechanism (Fulco *et al.* 2008, Canto *et al.* 2010). The circulating MNAM level can be regulated by the nutritional state in humans, i.e. fasting leads to an initial increase followed by a decline after a meal (Strom *et al.* 2018). However, exogenetic MNAM treatment did not stimulate calorie restriction, decreasing *Nampt* expression in skeletal muscle. Mechanisms involved in the negative regulation of *Nampt* by MNAM should be addressed in future experiments. HIF1α and Sod2 both protect cells from oxidative stress by limiting mitochondrial ROS levels, acting as key components in antioxidant defenses (Zhao *et al.* 2014, Janbandhu *et al.* 2022). Our data showed that MNAM treatment reversed the HFD-induced expression of *Hif1α* and *Sod2* in skeletal muscle. In addition, NAMPT increases resistance to oxidative stress in muscle cells by regulating sirtuin function (Garten *et al.* 2015). Thus, our data indicate that decreased *Nampt*, *Hif1α*, and *Sod2* all contribute to impaired antioxidant defenses in skeletal muscle, which in turn results in lower GLUT4, since the negative regulation of GLUT4 expression upon oxidative stress has been previously reported (Pessler *et al.* 2001).

Moreover, 1% MNAM supplementation reversed the HFD-induced higher gWAT mass. In line with this, increased expression of Sirt3 and decreased *Il1b* and *Il6* were found in gWAT of HM1% group at GD15.5. Sirt3 plays essential roles in mitochondrial function and has also been implicated in the regulation of inflammation in adipose tissue (Xu *et al.* 2016, Porter *et al.* 2018, Zhang *et al.* 2020). Elevated Hif1a in adipose tissue contributes to obesity-related chronic inflammation, insulin resistance, and metabolic dysfunction (Zhang *et al.* 2010). These data indicate that increased Sirt3 and decreased Hif1a may be involved in the alleviation effect of MNAM treatment in gWAT.

Besides the common metabolic organs, the placenta is involved in the pathophysiology of maternal glucose intolerance via placental hormones, which promote a mild state of insulin resistance to support the demands of the growing fetus (Radaelli *et al.* 2003, Bouchard *et al.* 2012). In contrast to WAT and skeletal muscle, insulin is not required for the placental transport of glucose. Instead, glucose transport occurs mainly via GLUT1 by carrier-mediated sodium-independent diffusion (Augustin 2010). In the current study, increased GLUT1 was observed in the placenta of the HFD group, which coordinates with higher maternal blood glucose, while MNAM prevented this adaptation in placenta. Interestingly, oxidative stress-related genes *Hif1α*, *Sod2*, and *Nrf2* were also induced in CM1% group, which may reflect placental glucose transport insufficiency (Myatt & Cui 2004). These findings suggest that induced* Hif1α*, *Sod2*, and *Nrf2* as well as weakened GLUT1 may contribute to the higher glucose levels in CM1% and HM1% groups, respectively. Notably, MNAM treatment in nonpregnant mice did not change either GTT. In summary, the placenta may be the major reason for the unexpected impaired GTT of maternal mice in the current study.

## Conclusion

As a major metabolite of vitamin B3 produced by NNMT, MNAM has been previously reported to exhibit an antidiabetic effect in male mice. In contrast, here we demonstrated that maternal MNAM treatment impaired glucose tolerance at mid-pregnancy in both CHOW- and HFD-fed mice by enhancing hepatic gluconeogenesis, lowering GLUT4 in skeletal muscle as well as reducing glucose transport in placenta, although ameliorating liver function and inflammation in adipose tissue. Our data provide new evidence for the role of MNAM in maternal glucose homeostasis and the careful usage of MNAM in GDM treatment.

## Supplementary materials

Supplementary Figures

## Declaration of interest

The authors declare that there is no conflict of interest that could be perceived as prejudicing the impartiality of the study reported.

## Funding

This work did not receive any specific grant from any funding agency in the public, commercial, or not-for-profit sector.

## Author contribution statement

Conceptualization, X.L.; methodology, X.W. and Y.T.; software, X.W. and Y.T.; validation, J.H. and X.D.; formal analysis, Z.Y.; investigation, Y.T., J.H., X.D., W. F. and T. L.; data curation, G.Y.; writing – original draft preparation, X.W.; writing – review and editing, X.L.; visualization, X.W.; supervision, X.L.; funding acquisition, X.W. and X.L. All authors have read and approved the final version of the manuscript.

## References

[bib1] Augustin R 2010 The protein family of glucose transport facilitators: it's not only about glucose after all. IUBMB Life 62 315–333. (10.1002/iub.315) 20209635

[bib2] Biedron R Ciszek M Tokarczyk M Bobek M Kurnyta M Slominska EM Smolenski RT & Marcinkiewicz J 2008 1-methylnicotinamide and nicotinamide: two related anti-inflammatory agents that differentially affect the functions of activated macrophages. Archivum Immunologiae et Therapiae Experimentalis 56 127–134. (10.1007/s00005-008-0009-2) 18373238 PMC2766500

[bib3] Bouchard L Hivert MF Guay SP St-Pierre J Perron P & Brisson D 2012 Placental adiponectin gene DNA methylation levels are associated with mothers' blood glucose concentration. Diabetes 61 1272–1280. (10.2337/db11-1160) 22396200 PMC3331769

[bib4] Brachs S Polack J Brachs M Jahn-Hofmann K Elvert R Pfenninger A Barenz F Margerie D Mai K Spranger J, *et al.* 2019 Genetic nicotinamide N-methyltransferase (Nnmt) deficiency in male mice improves insulin sensitivity in diet-induced obesity but does not affect glucose tolerance. Diabetes 68 527–542. (10.2337/db18-0780) 30552109

[bib5] Brown J Alwan NA West J Brown S McKinlay CJ Farrar D & Crowther CA 2017 Lifestyle interventions for the treatment of women with gestational diabetes. Cochrane Database of Systematic Reviews 5 CD011970. (10.1002/14651858.CD011970.pub2) 28472859 PMC6481373

[bib6] Buchanan TA Xiang AH & Page KA 2012 Gestational diabetes mellitus: risks and management during and after pregnancy. Nature Reviews Endocrinology 8 639–649. (10.1038/nrendo.2012.96) PMC4404707 22751341

[bib7] Canto C Jiang LQ Deshmukh AS Mataki C Coste A Lagouge M Zierath JR & Auwerx J 2010 Interdependence of AMPK and SIRT1 for metabolic adaptation to fasting and exercise in skeletal muscle. Cell Metabolism 11 213–219. (10.1016/j.cmet.2010.02.006) 20197054 PMC3616265

[bib8] Chlopicki S Swies J Mogielnicki A Buczko W Bartus M Lomnicka M Adamus J & Gebicki J 2007 1-methylnicotinamide (MNA), a primary metabolite of nicotinamide, exerts anti-thrombotic activity mediated by a cyclooxygenase-2/prostacyclin pathway. British Journal of Pharmacology 152 230–239. (10.1038/sj.bjp.0707383) 17641676 PMC1978255

[bib9] Ding Q Ma Y Lai S Dou X & Li S 2021 NNMT aggravates hepatic steatosis, but alleviates liver injury in alcoholic liver disease. Journal of Hepatology 74 1248–1250. (10.1016/j.jhep.2020.11.025) 33340581

[bib10] Fulco M Cen Y Zhao P Hoffman EP McBurney MW Sauve AA & Sartorelli V 2008 Glucose restriction inhibits skeletal myoblast differentiation by activating SIRT1 through AMPK-mediated regulation of Nampt. Developmental Cell 14 661–673. (10.1016/j.devcel.2008.02.004) 18477450 PMC2431467

[bib11] Garten A Schuster S Penke M Gorski T de Giorgis T & Kiess W 2015 Physiological and pathophysiological roles of NAMPT and NAD metabolism. Nature Reviews. Endocrinology 11 535–546. (10.1038/nrendo.2015.117) 26215259

[bib12] Hatting M Tavares CDJ Sharabi K Rines AK & Puigserver P 2018 Insulin regulation of gluconeogenesis. Annals of the New York Academy of Sciences 1411 21–35. (10.1111/nyas.13435) 28868790 PMC5927596

[bib13] Hong S Moreno-Navarrete JM Wei X Kikukawa Y Tzameli I Prasad D Lee Y Asara JM Fernandez-Real JM Maratos-Flier E, *et al.* 2015 Nicotinamide N-methyltransferase regulates hepatic nutrient metabolism through Sirt1 protein stabilization. Nature Medicine 21 887–894. (10.1038/nm.3882) PMC4529375 26168293

[bib14] Janbandhu V Tallapragada V Patrick R Li Y Abeygunawardena D Humphreys DT Martin EMMA Ward AO Contreras O Farbehi N, *et al.* 2022 Hif-1a suppresses ROS-induced proliferation of cardiac fibroblasts following myocardial infarction. Cell Stem Cell 29 281–297.e12. (10.1016/j.stem.2021.10.009) 34762860 PMC9021927

[bib15] Kannt A Pfenninger A Teichert L Tonjes A Dietrich A Schon MR Kloting N & Bluher M 2015 Association of nicotinamide-N-methyltransferase mRNA expression in human adipose tissue and the plasma concentration of its product, 1-methylnicotinamide, with insulin resistance. Diabetologia 58 799–808. (10.1007/s00125-014-3490-7) 25596852 PMC4351435

[bib16] Komatsu M Kanda T Urai H Kurokochi A Kitahama R Shigaki S Ono T Yukioka H Hasegawa K Tokuyama H, *et al.* 2018 NNMT activation can contribute to the development of fatty liver disease by modulating the NAD (+) metabolism. Scientific Reports 8 8637. (10.1038/s41598-018-26882-8) 29872122 PMC5988709

[bib17] Kraus D Yang Q Kong D Banks AS Zhang L Rodgers JT Pirinen E Pulinilkunnil TC Gong F Wang YC, *et al.* 2014 Nicotinamide N-methyltransferase knockdown protects against diet-induced obesity. Nature 508 258–262. (10.1038/nature13198) 24717514 PMC4107212

[bib18] Lee KW Ching SM Ramachandran V Yee A Hoo FK Chia YC Wan Sulaiman WA Suppiah S Mohamed MH & Veettil SK 2018 Prevalence and risk factors of gestational diabetes mellitus in Asia: a systematic review and meta-analysis. BMC Pregnancy and Childbirth 18 494. (10.1186/s12884-018-2131-4) 30547769 PMC6295048

[bib20] Li HY Liu YX Harvey L Shafaeizadeh S van der Beek EM & Han W 2020 A mouse model of gestation-specific transient hyperglycemia for translational studies. Journal of Endocrinology 244 501–510. (10.1530/JOE-19-0516) 31910155

[bib19] Li D Yi C Huang H Li J & Hong S 2022 Hepatocyte-specific depletion of Nnmt protects mice from non-alcoholic steatohepatitis. Journal of Hepatology 77 882–884. (10.1016/j.jhep.2022.03.021) 35367283

[bib21] Liu M Li L Chu J Zhu B Zhang Q Yin X Jiang W Dai G Ju W Wang Z, *et al.* 2015 Serum N(1)-Methylnicotinamide is associated with obesity and diabetes in Chinese. Journal of Clinical Endocrinology and Metabolism 100 3112–3117. (10.1210/jc.2015-1732) 26066674 PMC4525009

[bib22] Mateuszuk Ł Khomich TI Slominska E Gajda M Wojcik L Lomnicka M Gwozdz P & Chlopicki S 2009 Activation of nicotinamide N-methyltrasferase and increased formation of 1-methylnicotinamide (MNA) in atherosclerosis. Pharmacological Reports 61 76–85. (10.1016/s1734-1140(0970009-x) 19307695

[bib23] McIntyre HD Catalano P Zhang C Desoye G Mathiesen ER & Damm P 2019 Gestational diabetes mellitus. Nature Reviews. Disease Primers 5 47. (10.1038/s41572-019-0098-8) 31296866

[bib24] Mukherjee S Mo J Paolella LM Perry CE Toth J Hugo MM Chu Q Tong Q Chellappa K & Baur JA 2021 SIRT3 is required for liver regeneration but not for the beneficial effect of nicotinamide riboside. JCI Insight 6. (10.1172/jci.insight.147193) PMC8119200 33690226

[bib25] Myatt L & Cui X 2004 Oxidative stress in the placenta. Histochemistry and Cell Biology 122 369–382. (10.1007/s00418-004-0677-x) 15248072

[bib26] Nejabati HR Mihanfar A Pezeshkian M Fattahi A Latifi Z Safaie N Valiloo M Jodati AR & Nouri M 2018 N1-methylnicotinamide (MNAM) as a guardian of cardiovascular system. Journal of Cellular Physiology 233 6386–6394. (10.1002/jcp.26636) 29741779

[bib27] Nejabati HR Ghaffari-Novin M Fathi-Maroufi N Faridvand Y Holmberg HC Hansson O Nikanfar S & Nouri M 2022 N1-Methylnicotinamide: is it time to consider it as a dietary supplement for athletes? Current Pharmaceutical Design 28 800–805. (10.2174/1381612828666220211151204) 35152860

[bib28] Pessler D Rudich A & Bashan N 2001 Oxidative stress impairs nuclear proteins binding to the insulin responsive element in the GLUT4 promoter. Diabetologia 44 2156–2164. (10.1007/s001250100024) 11793016

[bib29] Pissios P 2017 Nicotinamide N-methyltransferase: more than a vitamin B3 clearance enzyme. Trends in Endocrinology and Metabolism 28 340–353. (10.1016/j.tem.2017.02.004) 28291578 PMC5446048

[bib30] Plows JF Stanley JL Baker PN Reynolds CM & Vickers MH 2018 The pathophysiology of gestational diabetes mellitus. International Journal of Molecular Sciences 19. (10.3390/ijms19113342) PMC6274679 30373146

[bib31] Porter LC Franczyk MP Pietka T Yamaguchi S Lin JB Sasaki Y Verdin E Apte RS & Yoshino J 2018 NAD(+)-dependent deacetylase SIRT3 in adipocytes is dispensable for maintaining normal adipose tissue mitochondrial function and whole body metabolism. American Journal of Physiology. Endocrinology and Metabolism 315 E520–E530. (10.1152/ajpendo.00057.2018) 29634313 PMC6230701

[bib32] Radaelli T Varastehpour A Catalano P & Hauguel-de Mouzon S 2003 Gestational diabetes induces placental genes for chronic stress and inflammatory pathways. Diabetes 52 2951–2958. (10.2337/diabetes.52.12.2951) 14633856

[bib33] Rasmussen L Poulsen CW Kampmann U Smedegaard SB Ovesen PG & Fuglsang J 2020 Diet and healthy lifestyle in the management of gestational diabetes mellitus. Nutrients 12. (10.3390/nu12103050) PMC7599681 33036170

[bib34] Salek RM Maguire ML Bentley E Rubtsov DV Hough T Cheeseman M Nunez D Sweatman BC Haselden JN Cox RD, *et al.* 2007 A metabolomic comparison of urinary changes in type 2 diabetes in mouse, rat, and human. Physiological Genomics 29 99–108. (10.1152/physiolgenomics.00194.2006) 17190852

[bib35] Song Q Chen Y Wang J Hao L Huang C Griffiths A Sun Z Zhou Z & Song Z 2020 ER stress-induced upregulation of NNMT contributes to alcohol-related fatty liver development. Journal of Hepatology 73 783–793. (10.1016/j.jhep.2020.04.038) 32389809 PMC8301603

[bib36] Sternak M Khomich TI Jakubowski A Szafarz M Szczepanski W Bialas M Stojak M Szymura-Oleksiak J & Chlopicki S 2010 Nicotinamide N-methyltransferase (NNMT) and 1-methylnicotinamide (MNA) in experimental hepatitis induced by concanavalin A in the mouse. Pharmacological Reports 62 483–493. (10.1016/s1734-1140(1070304-2) 20631412

[bib37] Strom K Morales-Alamo D Ottosson F Edlund A Hjort L Jorgensen SW Almgren P Zhou Y Martin-Rincon M Ekman C, *et al.* 2018 N1-methylnicotinamide is a signalling molecule produced in skeletal muscle coordinating energy metabolism. Scientific Reports 8 3016. (10.1038/s41598-018-21099-1) 29445118 PMC5813101

[bib38] Vandesompele J De Preter K Pattyn F Poppe B Van Roy N De Paepe A & Speleman F 2002 Accurate normalization of real-time quantitative RT-PCR data by geometric averaging of multiple internal control genes. Genome Biology 3 RESEARCH0034. (10.1186/gb-2002-3-7-research0034) 12184808 PMC126239

[bib39] Wang H Li N Chivese T Werfalli M Sun H Yuen L Hoegfeldt CA Elise Powe C Immanuel J Karuranga S, *et al.* 2022 IDF diabetes atlas: estimation of global and regional gestational diabetes mellitus prevalence for 2021 by international association of diabetes in pregnancy study Group's criteria. Diabetes Research and Clinical Practice 183 109050. (10.1016/j.diabres.2021.109050) 34883186

[bib40] Watala C Kazmierczak P Dobaczewski M Przygodzki T Bartus M Lomnicka M Slominska EM Durackova Z & Chlopicki S 2009 Anti-diabetic effects of 1-methylnicotinamide (MNA) in streptozocin-induced diabetes in rats. Pharmacological Reports 61 86–98. (10.1016/s1734-1140(0970010-6) 19307696

[bib41] Wei X Jia R Wang G Hong S Song L Sun B Chen K Wang N Wang Q Luo X, *et al.* 2020 Depot-specific regulation of NAD(+)/SIRTs metabolism identified in adipose tissue of mice in response to high-fat diet feeding or calorie restriction. Journal of Nutritional Biochemistry 80 108377. (10.1016/j.jnutbio.2020.108377) 32278117

[bib42] Wei X Wei C Tan Y Dong X Yang Z Yan J & Luo X 2023 Both prolonged high-fat diet consumption and calorie restriction boost hepatic NAD^+^ metabolism in mice. Journal of Nutritional Biochemistry 115 109296. (10.1016/j.jnutbio.2023.109296) 36849030

[bib43] Xu H Hertzel AV Steen KA & Bernlohr DA 2016 Loss of fatty acid binding protein 4/aP2 reduces macrophage inflammation through activation of SIRT3. Molecular Endocrinology 30 325–334. (10.1210/me.2015-1301) 26789108 PMC4771695

[bib44] Xu L Yang C Ma J Zhang X Wang Q & Xiong X 2021 NAMPT-mediated NAD(+) biosynthesis suppresses activation of hepatic stellate cells and protects against CCl(4)-induced liver fibrosis in mice. Human and Experimental Toxicology 40 S666–S675. (10.1177/09603271211052991) 34752167

[bib45] Zhang X Lam KSL Ye H Chung SK Zhou M Wang Y & Xu A 2010 Adipose tissue-specific inhibition of hypoxia-inducible factor 1alpha induces obesity and glucose intolerance by impeding energy expenditure in mice. Journal of Biological Chemistry 285 32869–32877. (10.1074/jbc.M110.135509) 20716529 PMC2963410

[bib46] Zhang J Chen Y Liu C Li L & Li P 2020 N1-Methylnicotinamide Improves Hepatic Insulin Sensitivity via Activation of SIRT1 and Inhibition of FOXO1 Acetylation. Journal of Diabetes Research 2020 1080152. (10.1155/2020/1080152) 32280711 PMC7125486

[bib47] Zhao T Zhu Y Morinibu A Kobayashi M Shinomiya K Itasaka S Yoshimura M Guo G Hiraoka M & Harada H 2014 HIF-1-mediated metabolic reprogramming reduces ROS levels and facilitates the metastatic colonization of cancers in lungs. Scientific Reports 4 3793. (10.1038/srep03793) 24452734 PMC3899644

[bib48] Zhuang A Yang C Liu Y Tan Y Bond ST Walker S Sikora T Laskowski A Sharma A de Haan JB, *et al.* 2021 SOD2 in skeletal muscle: new insights from an inducible deletion model. Redox Biology 47 102135. (10.1016/j.redox.2021.102135) 34598016 PMC8487078

