# Supplementary material for: N1-methylnicotinamide impairs gestational glucose tolerance in mice
Source: J Mol Endocrinol. 2024 Jan 8;72(2):e230126. doi: 10.1530/JME-23-0126 (PMC10831565; doi:10.1530/JME-23-0126)
Supplement: Supplementary Figures [file supplementary_figures.pdf]

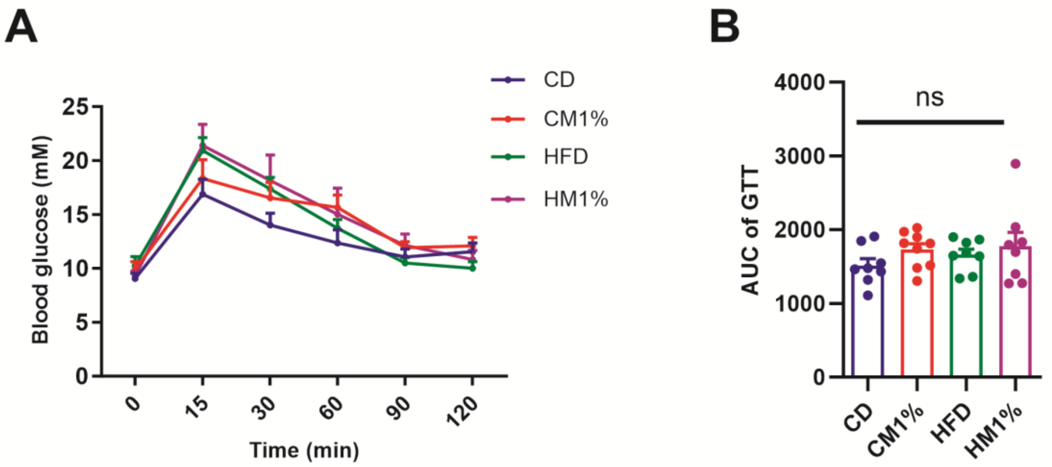

Figure S1 Glucose tolerance test in non-pregnant mice challenged with HFD for 6 weeks followed by MNAM treatment for 2 weeks (A) and AUC of glucose levels (B). Data are presented as mean ± SEM. CD, n=8; CM1%, n=9; HFD, n=8; HM1%; n=8. Statistical significance was tested by one-way ANOVA followed by Tukey's post hoc tests. "ns" is not significant

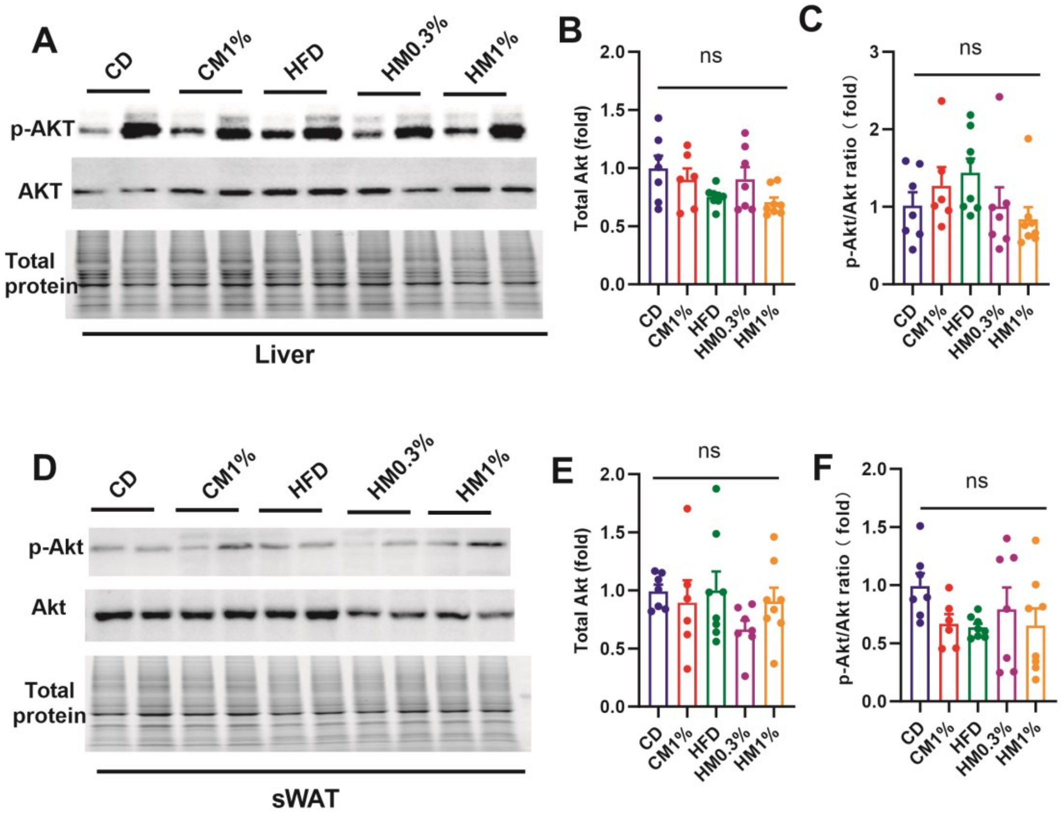

Figure S2 Phosphorylation of AKT in liver and sWAT collected at GD18.5. (**A, B, C**) Representative Western blots of total and phosphorylated Akt (S473) in liver and quantification of total Akt and p-Akt/Akt ratio in liver. (**D, E, F**) Representative Western blots of total and phosphorylated Akt (S473) and quantification in sWAT. Total protein was set as loading control. Data are presented as mean ± SEM. CD, n=7; CM1%, n=6; HFD, n=8; HM0.3%, n=7; HM1%; n=8. Statistical significance was tested by one-way ANOVA followed by Tukey's post hoc tests.

"ns" is not significant

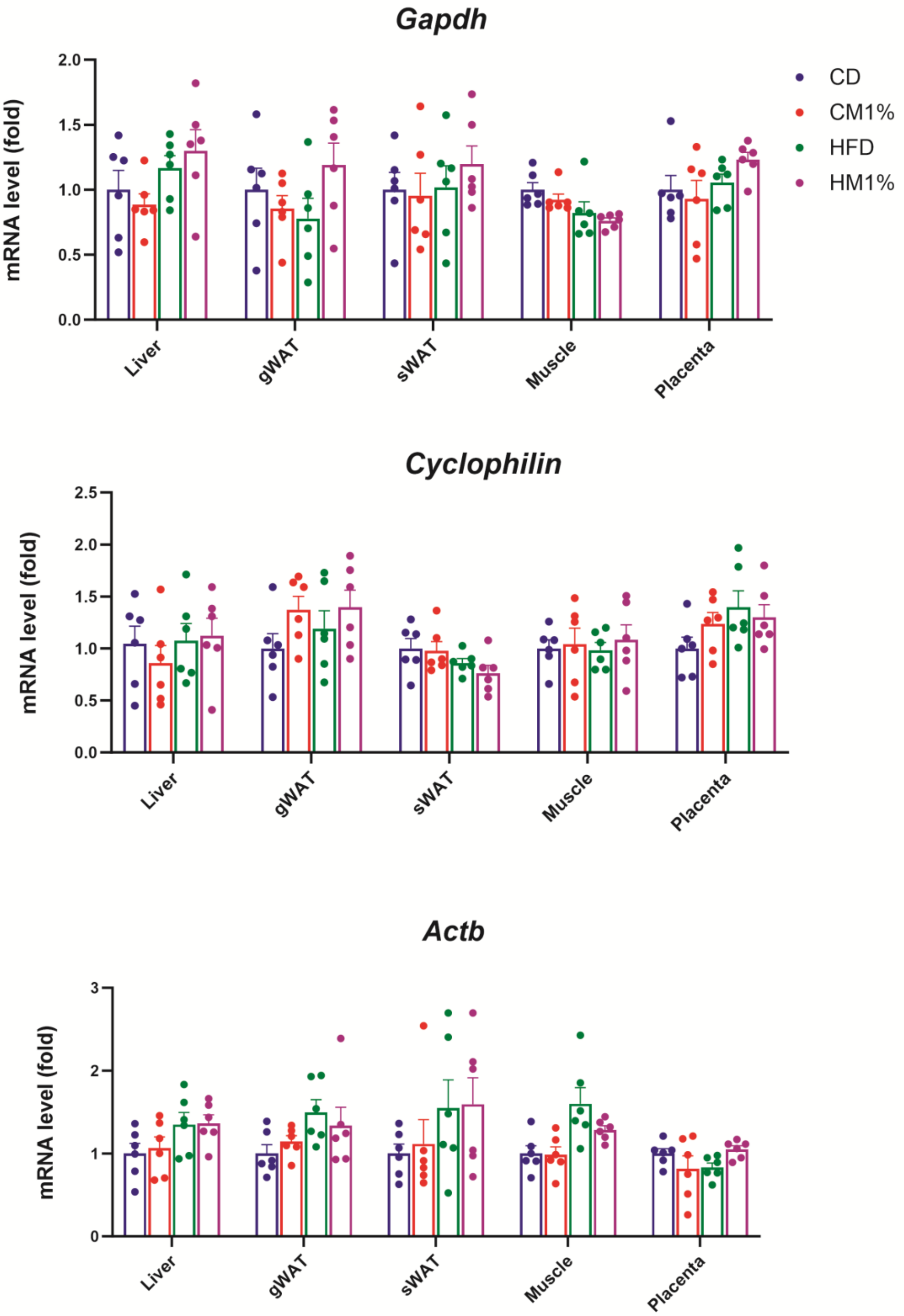

Figure S3 Relative gene expression of Gapdh, Cyclophilin and Actb in liver, gWAT, sWAT, skeletal muscle and placenta on GD15.5. Data are presented as mean ± SEM and normalized to CD group. n=6/group.

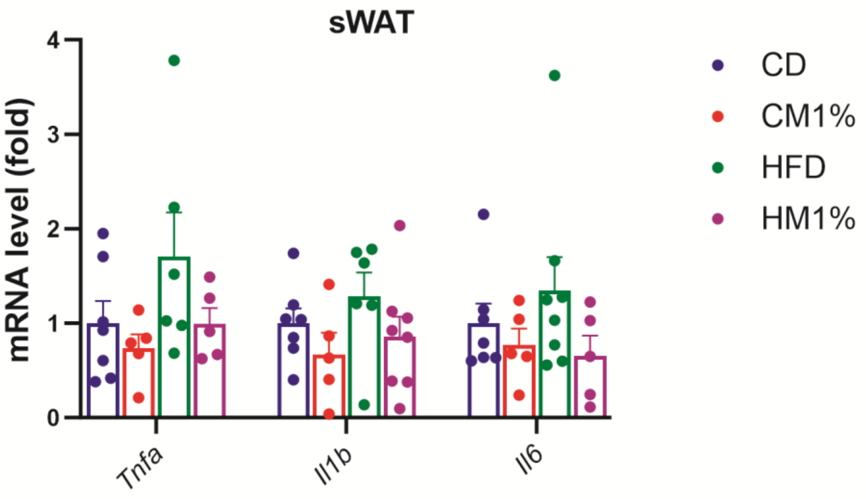

Figure S4 Relative mRNA expression of inflammatory genes in sWAT. Data are presented as mean ± SEM. CD, n=7; CM1%, n=5; HFD, n=6; HM1%; n=8. TNFα, Tumor necrosis factor alpha; Il1b, interleukin 1 β; Il6, interleukin. Expression data were calculated by geometric averaging of multiple internal control genes, Gapdh, Cyclophilin and Actb. sWAT, inguinal subcutaneous WAT.

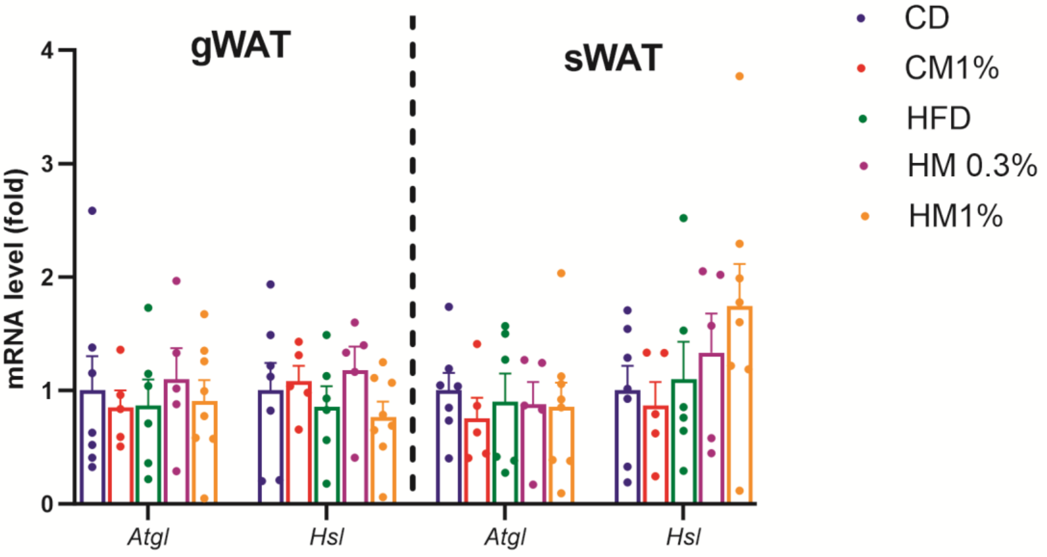

Figure S5 Relative mRNA expression of key enzymes in lipolysis in adipose tissue. Data are presented as mean ± SEM. CD, n=7; CM1%, n=5; HFD, n=6; HM0.3%, n=5; HM1%; n=8 ATGL, adipose triglyceride lipase; hormone sensitive lipase. Expression data were calculated by geometric averaging of multiple internal control genes, *Gapdh*, *Cyclophilin* and *Actb*. gWAT, gonadal white adipose tissue; sWAT, inguinal subcutaneous WAT.

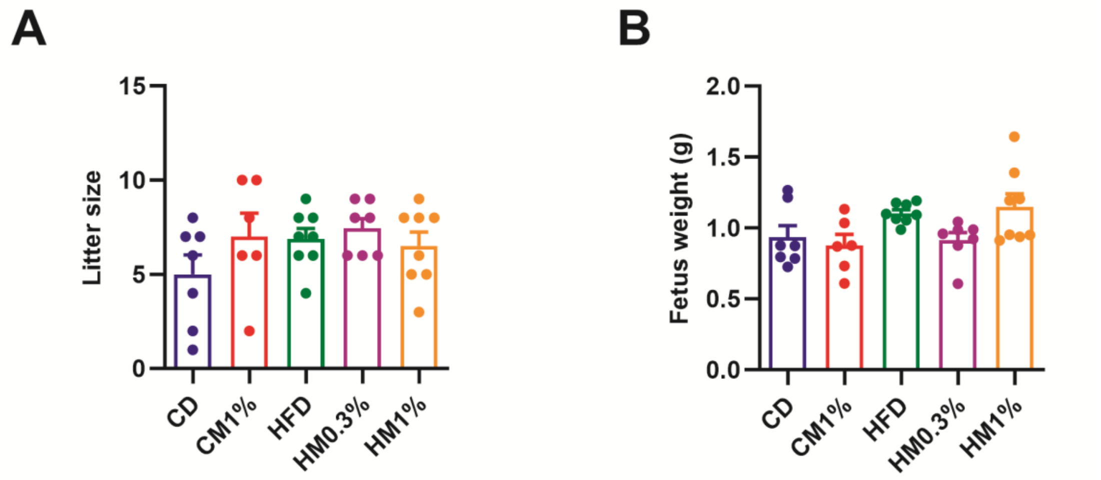

Figure S6 Litter size (A) and fetus weight (B) measured after cesarean section at GD18.5. Data are presented as mean ± SEM. CD, n=7; CM1%, n=6; HFD, n=8; HM0.3%, n=7; HM1%; n=8.
